# Spontaneous coronary artery dissection in a woman taking phytoestrogens

**DOI:** 10.1093/ehjcr/ytaf596

**Published:** 2025-11-18

**Authors:** Marcos Albarrán, Manuel Martínez-Sellés

**Affiliations:** Cardiology Department, Hospital General Universitario Gregorio Marañón, Instituto de Investigación Sanitaria Hospital General Universitario Gregorio Marañón, Cardiovascular Research Center (CIBERCV), Instituto de Salud Carlos III, Calle Doctor Esquerdo, 46, Madrid 28007, Spain; Cardiology Department, Hospital General Universitario Gregorio Marañón, Instituto de Investigación Sanitaria Hospital General Universitario Gregorio Marañón, Cardiovascular Research Center (CIBERCV), Instituto de Salud Carlos III, Calle Doctor Esquerdo, 46, Madrid 28007, Spain; School of Biomedical and Health Sciences, Universidad Europea, School of Medicine, Universidad Complutense, C. Tajo, s/n, 28670 Villaviciosa de Odón, Madrid, Spain

## Case description

Spontaneous coronary artery dissection (SCAD) accounts for 25%–35% myocardial infarctions in middle-aged women and has been associated with hyperestrogenism.^1,2^ Phytoestrogens are plant compounds with a chemical structure similar to 17β-estradiol, capable of binding to oestrogen receptors.^3^

**Figure 1:**
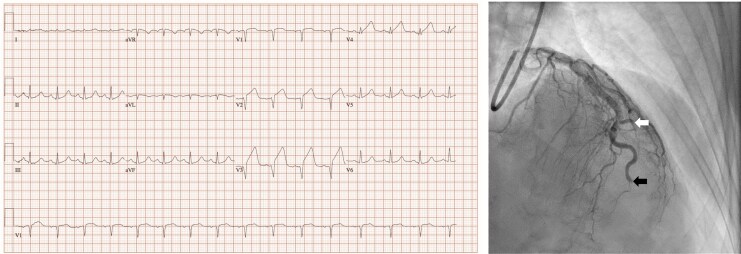
ECG showing ST-segment elevation in anterior leads and coronary angiography with left anterior descending artery spontaneous coronary artery dissection with distal occlusion (black arrow) and occlusion of the first diagonal artery (white arrow).

A 62-year-old woman had been receiving for 6 months MenoMaster, a product containing soy isoflavones, used to alleviate menopausal symptoms. She developed chest pain and arrived at our centre 45 min after symptom onset. ECG in the emergency department showed ST-segment elevation in anterior leads and coronary angiography (door-to-angiography time: 35 min) depicted left anterior descending artery SCAD with distal occlusion and occlusion of the first diagonal artery (*Figure 1*). Due to ongoing chest pain and ST-segment elevation, revascularization was attempted but was unsuccessful. After 90 min of hospital arrival, the patient was asymptomatic, with 45% left ventricular ejection fraction in echocardiography. We ruled out other potential causes of SCAD (stress, fibromuscular dysplasia, connective tissue disorders, drug use). The patient was discharged on Day 8 with 100 mg aspirin and recommendation to discontinue MenoMaster.

SCAD is a frequent cause of acute myocardial infarction in women.^1^ The risk increases with hormone replacement therapy and during pregnancy.^2^ The predominant hypothesis is that oestrogen excess may induce structural changes in the extracellular matrix of the arterial wall, promoting collagen degeneration, medial weakening, and intramural haematoma formation. Exogenous oestrogens, from sources like oral contraceptives or hormone therapy, may contribute to SCAD by promoting vascular changes such as increased metalloproteinase activity, which weakens the arterial wall. Soy isoflavones, due to their affinity for β-oestrogen receptors, may exert similar vascular effects. In our case, the concomitance of prolonged phytoestrogen intake and SCAD raises the possibility of a facilitating effect, although a direct causal relationship cannot be established. In any case, it seems prudent to recommend discontinuation of phytoestrogen supplements after SCAD.

## Supplementary Material

ytaf596_Supplementary_Data

## Data Availability

All data are incorporated into the article and its online Supplementary material.

## Notes

**Consent:** The patient signed the European Heart Journal—Case Reports Patient Consent Form Spanish translation.

**Funding:** None declared.
